# ME-NBI combined with endoscopic ultrasonography for diagnosing and staging the invasion depth of early esophageal cancer: a diagnostic meta-analysis

**DOI:** 10.1186/s12957-022-02809-6

**Published:** 2022-10-17

**Authors:** Feng Su, Meiling Zhu, Ru Feng, Yunhong Li

**Affiliations:** Department of Gastroenterology, The Affiliated Suqian Hospital of Xuzhou Medical University, Suqian Hospital of Nanjing Drum Tower Hospital Group, Suqian, Jiangsu Province 223800 China

**Keywords:** Magnifying endoscopy, Narrow band imaging, Endoscopic ultrasonography, Early esophageal cancer, Meta-analysis

## Abstract

**Background:**

Several methods can assist in detecting early esophageal cancer (EEC) and staging esophageal cancer (EC) invasion depth.

**Objective:**

To evaluate the accuracy of magnifying endoscopy with narrow-band imaging (ME-NBI) plus endoscopic ultrasonography (EUS) for diagnosing EC.

**Methods:**

We searched the PubMed, Embase, Cochrane Library, and China National Knowledge Infrastructure (CNKI) databases for relevant studies. The Quality Assessment of Diagnostic Accuracy Studies 2 (QADAS2) was used to assess the studies’ methodological quality. The sensitivity, specificity, positive likelihood (LR+), negative likelihood (LR−), and diagnostic odds ratio (DOR) were calculated, and the summary receiver operating characteristic (SROC) curves were drawn to evaluate the diagnostic performance.

**Results:**

Seven studies were included. The meta-analysis suggested that the pooled sensitivity, specificity, LR+, LR−, and DOR of ME-NBI plus EUS for diagnosing EC were 0.947 (95% confidence interval [CI], 0.901–0.975), 0.894 (95% *CI*, 0.847–0.931), 7.989 (95% *CI*, 4.264–14.970), 0.066 (95% *CI*, 0.035–0.124), and 137.96 (95% *CI*, 60.369–315.27), respectively. Those values for staging the invasive depth were 0.791 (95% *CI*, 0.674–0.881), 0.943 (95% *CI*, 0.906–0.968), 13.087 (95% *CI*, 7.559–22.657), 0.226 (95% *CI*, 0.142–0.360), and 61.332 (95% *CI*, 27.343–137.57). The areas under the curves (AUCs) for diagnosis and staging were 0.97 and 0.95, respectively.

**Conclusions:**

ME-NBI plus EUS might be an adequate diagnostic and staging modality for EC. Due to the study limitations, more large-scale, high-quality studies are needed to confirm the diagnostic accuracy of ME-NBI plus EUS.

**Supplementary Information:**

The online version contains supplementary material available at 10.1186/s12957-022-02809-6.

## Introduction

Esophageal cancer (EC) is one of the most common malignancies of the digestive tract [1] and ranks seventh in new cases and sixth in cancer-related deaths worldwide [2]. Advanced EC has an extremely poor prognosis, with a 5-year survival rate of less than 10% [3]. The depth of invasion and lymph node metastasis are directly associated with EC prognosis [4]. Therefore, it is critically important to determine whether EC invasion is still limited to the mucosal membrane or submucosa without lymph node metastasis [5–7], because early esophageal cancer (EEC) has a better prognosis [4].

Several endoscopic modalities for the early detection of EEC and staging of the invasion depth of EEC, such as magnifying endoscopy (ME) with narrow-band imaging (NBI) [8], chromoscopy (e.g., Lugol staining) [9], and endoscopic ultrasound (EUS) [10] have been introduced. Several systematic reviews and meta-analyses reported the diagnostic accuracy of ME-NBI and EUS in EEC [8, 10, 11]. Moreover, a systematic review and meta-analysis with subgroup analysis have been performed to investigate the diagnostic accuracy of ME-NBI and EUS separately and confirmed that ME-NBI might be an optimal modality for the early detection of EEC [11]. Nevertheless, the diagnostic yield of individual modalities introduced previously is limited due to early detection and accurate differentiation of cancer infiltration.

Previous studies investigated the diagnostic accuracy of ME-NBI combined with EUS to improve the diagnostic yield of individual endoscopic modalities in EEC [12, 13], but a definitive conclusion has not yet been reached. Therefore, the present systematic review and meta-analysis were conducted to investigate the pooled diagnostic accuracy of ME-NBI combined with EUS in timely diagnosing and accurately differentiating the invasion depth of EEC.

## Methods

### Study design

We developed the framework of the current diagnostic meta-analysis according to the recommendations issued by the Cochrane Collaboration [14] to ensure methodological quality because no formal protocol was registered. Moreover, we followed the Preferred Reporting Items for a Systematic Review and Meta-analysis of Diagnostic Test Accuracy (PRISMA-DTA) [15] guidelines to report all the pooled results. The study did not require ethical approval and patient’s informed consent, as all essential data in the current diagnostic meta-analysis were extracted from published studies.

### Identification of the studies

We systematically searched the PubMed, Embase, Cochrane Library, and China National Knowledge Infrastructure (CNKI) databases to identify potentially eligible records. The search time was initially limited from database inception to July 2020, but the latest literature retrieval was performed on May 31, 2022. We developed the search strategy using the combination of medical subject headings (MeSH) and text words and modified it to meet the unique requirements of each database. All search strategies are presented in Tables S1, S2, S3 and S4. Meanwhile, we manually checked the reference lists of all included studies and topic-related reviews to identify additional studies. Only studies published in English or Chinese were included because no translators with expertise in other languages were enrolled. Any disagreements regarding the identification of studies were resolved through discussion or consulting a third senior reviewer.

### Selection criteria

We selected eligible studies according to the following criteria: (a) patients who received white light endoscopy (WLE) examination and were suspected of having EEC or precancerous lesions in the esophagus, (b) patients who were assigned to receive ME-NBI plus EUS for the diagnosis and staging of EEC, (c) the pathological results of all specimens from suspected lesions were determined by histological evaluation, (d) only retrospective or prospective studies that reported sufficient data were considered for inclusion, and (e) in the presence of multiple reports from the same dataset or patient cohort, only the study with the most information was considered.

The studies with one or more of the following criteria were excluded: (a) case reports and case series, (b) insufficient data to reconstruct a diagnostic 2 × 2 table, (c) no eligible outcomes and no additional information were added after contacting the lead author, and (d) in vivo or in vitro studies. Any disagreements about the selection of studies were solved through discussion or consulting a third senior reviewer.

### Outcomes of interest

The pooled sensitivity, specificity, positive likelihood ratio (LR+), negative likelihood ratio (LR−), diagnostic odds ratio (DOR), and summary receiver operating characteristic (SROC) curve of ME-NBI plus EUS for EEC were the primary outcomes. The secondary outcomes were the pooled sensitivity, specificity, LR+, LR−, DOR, and SROC curve of ME-NBI plus EUS for staging the invasion depth of EEC.

### Data extraction

The data extraction sheet was designed according to the aim and selection criteria of the study. Two independent investigators were assigned to accurately extract all essential information, including the first author, publication year, country of the corresponding author, sex, age, sample size, number of lesions, equipment of gastroscopy and EUS, outcomes, and risk of bias. Any disagreements about data extraction were resolved through discussion or consulting a third senior reviewer.

### Assessment of study quality

The methodological quality of an individual study was associated with the reliability and robustness of the results. Two independent investigators assessed the quality of all included studies using the Quality Assessment of Diagnostic Accuracy Studies 2 (QADAS2) tool [16]. All assessment results were cross-checked, and any divergences were resolved through discussion or consulting a third senior reviewer.

### Statistical analysis

The results of the combination of ME-NBI plus EUS were compared to the results of the final histopathological evaluation. Then, 2 × 2 numerical tables that included the true-positive (TP), false-positive (FP), true-negative (TN), and false-negative (FN) values were constructed. When one cell did not contain data, the continuity correction method was used to insert 0.5 [11]. After preparing the essential data, the Meta-Disc 1.4 software was used to calculate the sensitivity, specificity, LR+, LR−, and DOR [17] and the pooled sensitivity, specificity, LR+, LR−, and DOR [18]. STATA 14.0 was used to construct the summary receiver operating characteristic curve (SROC) and obtain the area under the curve (AUC) [19], which is a diagnostic indicator of performance. A test with an AUC close to 1 indicated excellent diagnostic yield, whereas a test with an AUC close to 0.5 indicated poor diagnostic yield [20]. In addition, several indicators, including chi-square statistic, Cochran’s Q, and *I*^2^ statistic, were estimated to qualitatively or quantitatively evaluate the heterogeneity across studies [21–23]. STATA 14.0 software was used to draw the funnel plots to inspect publication bias [24–26]. Sensitivity analysis was conducted to test the robustness of the pooled results when heterogeneity was detected. A *P* of less than 0.05 indicated statistical significance.

## Results

### Literature search

**Fig. 1:**
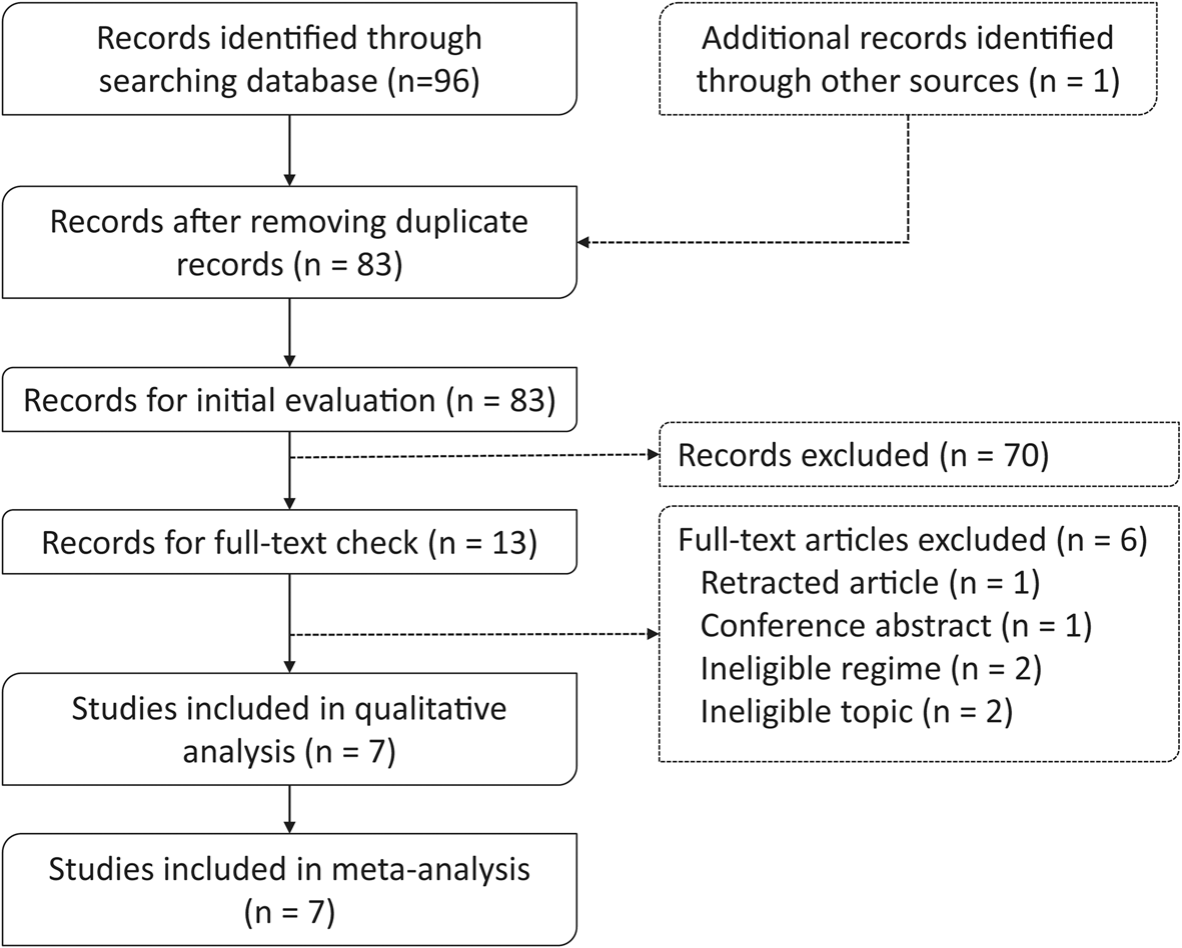
Flow diagram of study retrieval and selection

The initial search of the four databases identified 96 records. After removing the duplicates, 83 unique records were included for initial evaluation. The full texts of 12 studies were evaluated after excluding 70 ineligible records by checking the title, abstract, and keywords. Six studies that met the inclusion criteria were included after excluding six ineligible studies due to the following reasons: retracted article (*n* = 1), conference abstract (*n* = 1), ineligible intervention regime (*n* = 1), and unrelated to topic (*n* = 1). Moreover, an additional study [27] was added after reviewing the six eligible studies. Finally, seven eligible studies were included [12, 13, 27–30]. The identification and selection process of eligible studies is presented in Fig. 1.

### Characteristics of included studies

After critically checking all retrieved records, seven eligible studies [12, 13, 27–30] were included in the final analysis. All eligible studies were published in China, and the publication year ranged from 2009 to 2019. Of these seven studies, three [12, 27, 28] reported the diagnostic accuracy of ME-NBI combined with NBI in the early detection of EEC, and three [13, 29, 30] reported the value of ME-NBI combined with NBI in accurately differentiating the invasion depth of EEC. The remaining study [13] reported the diagnostic accuracy of ME-NBI combined with NBI for the early diagnosis and staging of invasion depth. The sample size of the individual studies ranged between 57 and 132, with a median of 95. Six studies [12, 13, 27–30] reported the details of endoscopic and EUS equipment, except for one study [13]. The detailed characteristics of the included studies are presented in Table 1.

**Table 1:** Characteristics of all 7 included studies in the present meta-analysis

**Study**	**Male (%)**	**Age**	**Sample**	**No. of lesions**	**Endoscopic equipment**	**EUS**	**TP**	**FP**	**FN**	**TN**
Diagnosis										
Wang 2009 [12]	60.3	20~81	57	57	Olympus GIF-Q260	UM-2000	22	8	1	26
Liu 2018 [27]	71.8	66.8 ± 7.9	85	88	GIF-Q260/Q260	EU-ME1	62	2	2	22
Liu 2017 [28]	62.1	33~82	132	144	GIF-H260Z	UM-2000	29	7	3	105
Su 2019^a^ [13]	52.1	56.9 ± 7.8	94	107	GIF-H260Z	FujinonSP 702	47	7	3	50
Staging										
Zheng 2019a [31]	59	45~76	105	105	n.r.	n.r.	27	5	6	67
Zheng 2019b [29]	70.7	61.04 ± 7.14	99	71	GIF-H260Z	FUJIFILM SU-8000	7	5	2	57
Su 2019^a^ [13]	52.1	56.9 ± 7.8	94	107	GIF-H260Z	FujinonSP 702	9	2	2	28
Xie 2019 [29]	74.7	45~77	95	95	GIF-H260Z/GIF-HQ290	UM-DP12/20-25R	10	2	4	79

*EUS* Endoscopic ultrasonography, *TP* True positive, *FP* False positive, *FN* False negative, *TN* True negative, *n.r.* Not reported

^a^The same study

### Quality of the eligible studies

**Fig. 2:**
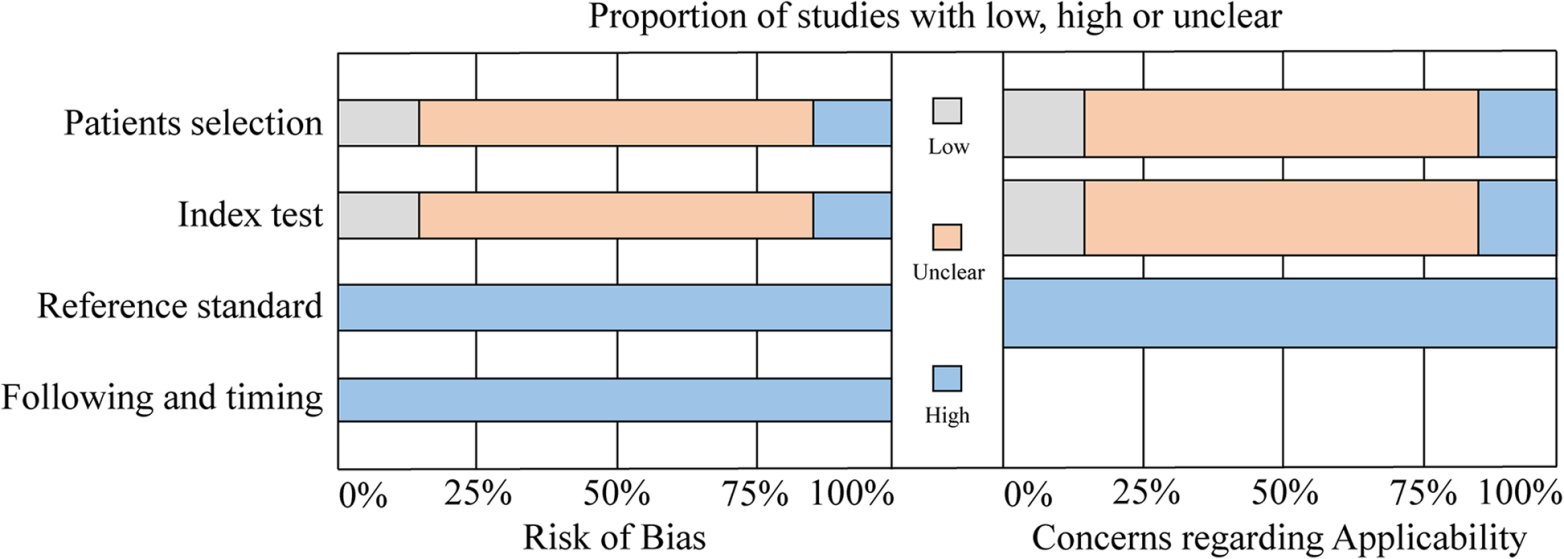
The quality of eligible studies according to the Diagnostic Accuracy Studies 2 tool

The quality of the individual study was assessed with QUADAS 2. One study [12] was labeled at high risk of bias in patient selection and index tests. All other included studies were labeled at low risk of bias compared to the reference standard and flow and timing. For applicability concerns, one study [12] had a high risk of bias in patient selection and index tests, and two studies [28, 30] had a low risk in these two items. These results suggested an overall quality of low to moderate level, and the summary of the quality evaluation is depicted in Fig. 2.

### Meta-analysis of early diagnosis

**Fig. 3:**
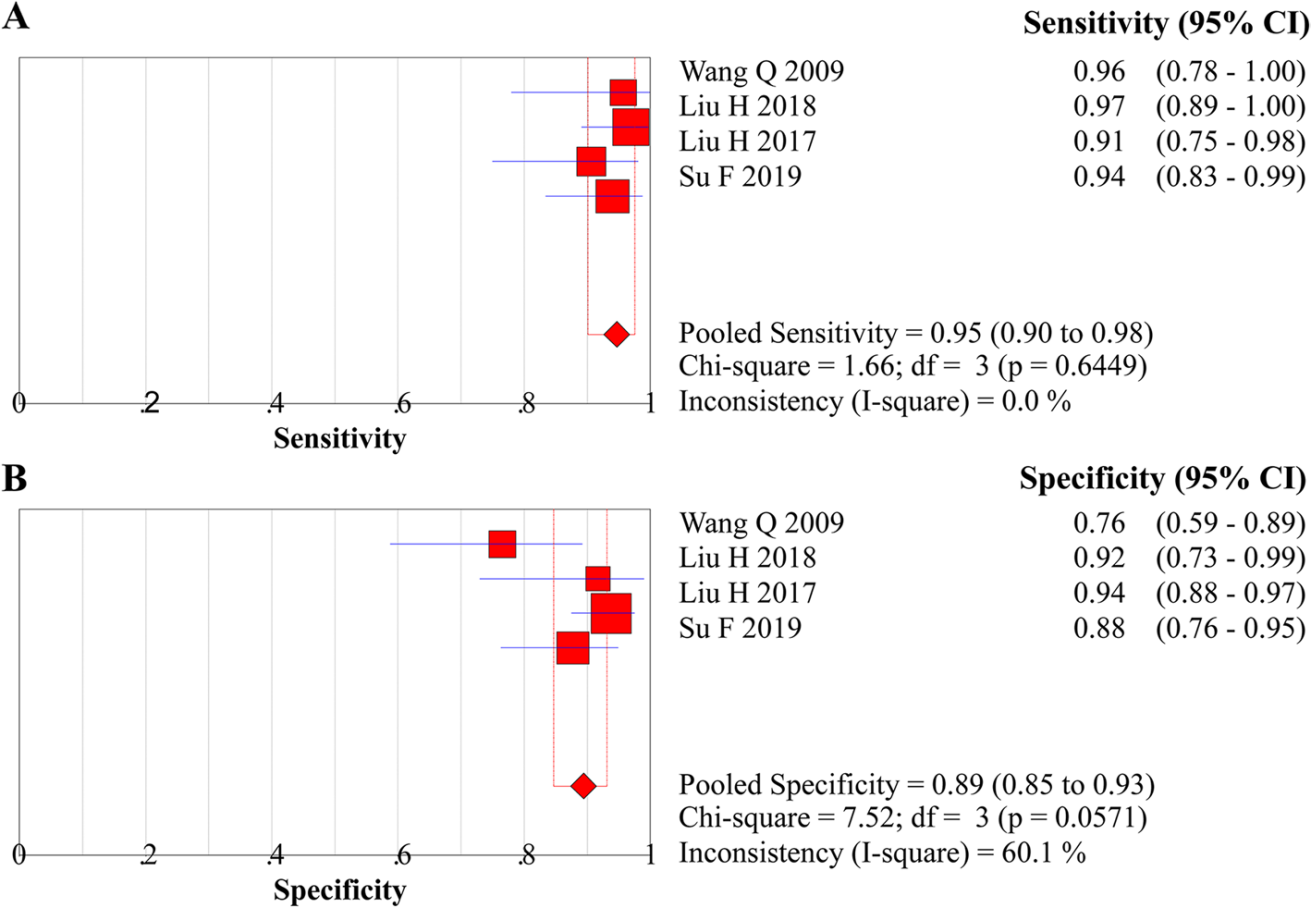
**A** Forest plot of sensitivity for diagnosing EEC. **B** Forest plot of specificity for diagnosing EEC

**Fig. 4:**
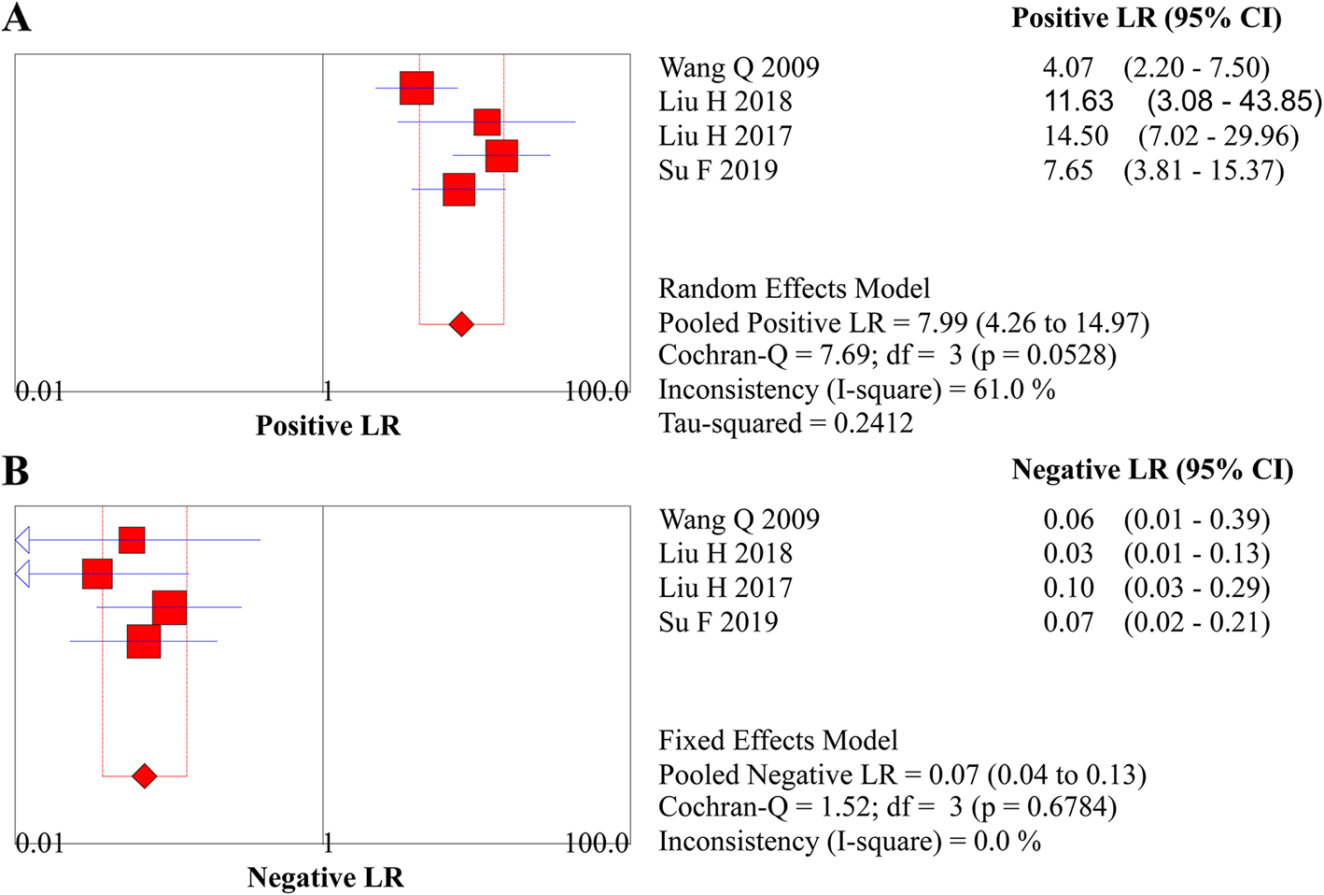
**A** Forest plot of LR+ for diagnosing EEC. **B** Forest plot of LR− for diagnosing EEC

**Fig. 5:**
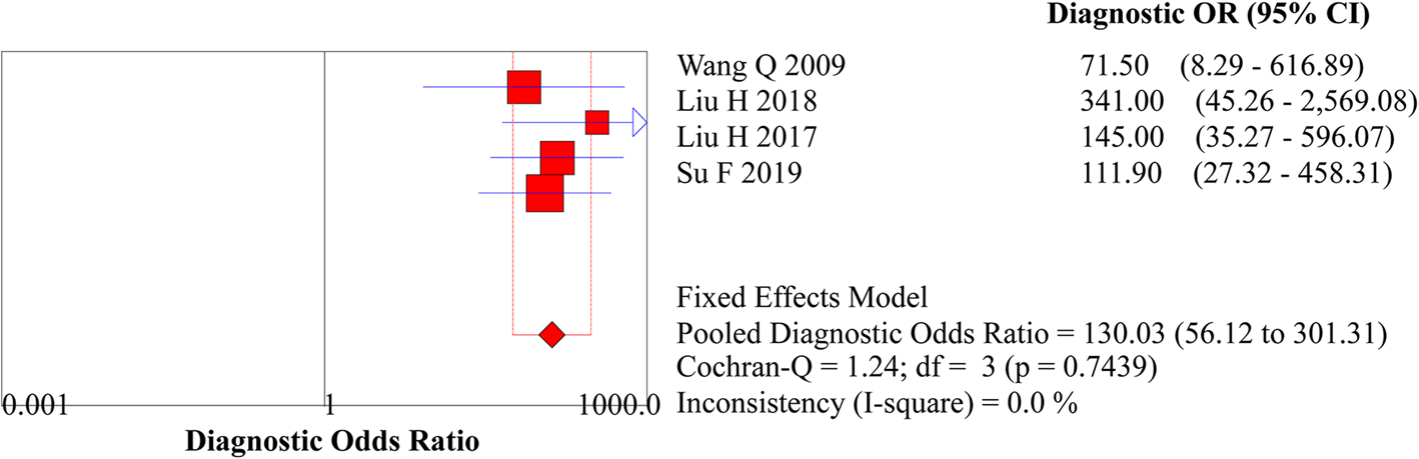
Forest plot of DOR for diagnosing EEC

**Fig. 6:**
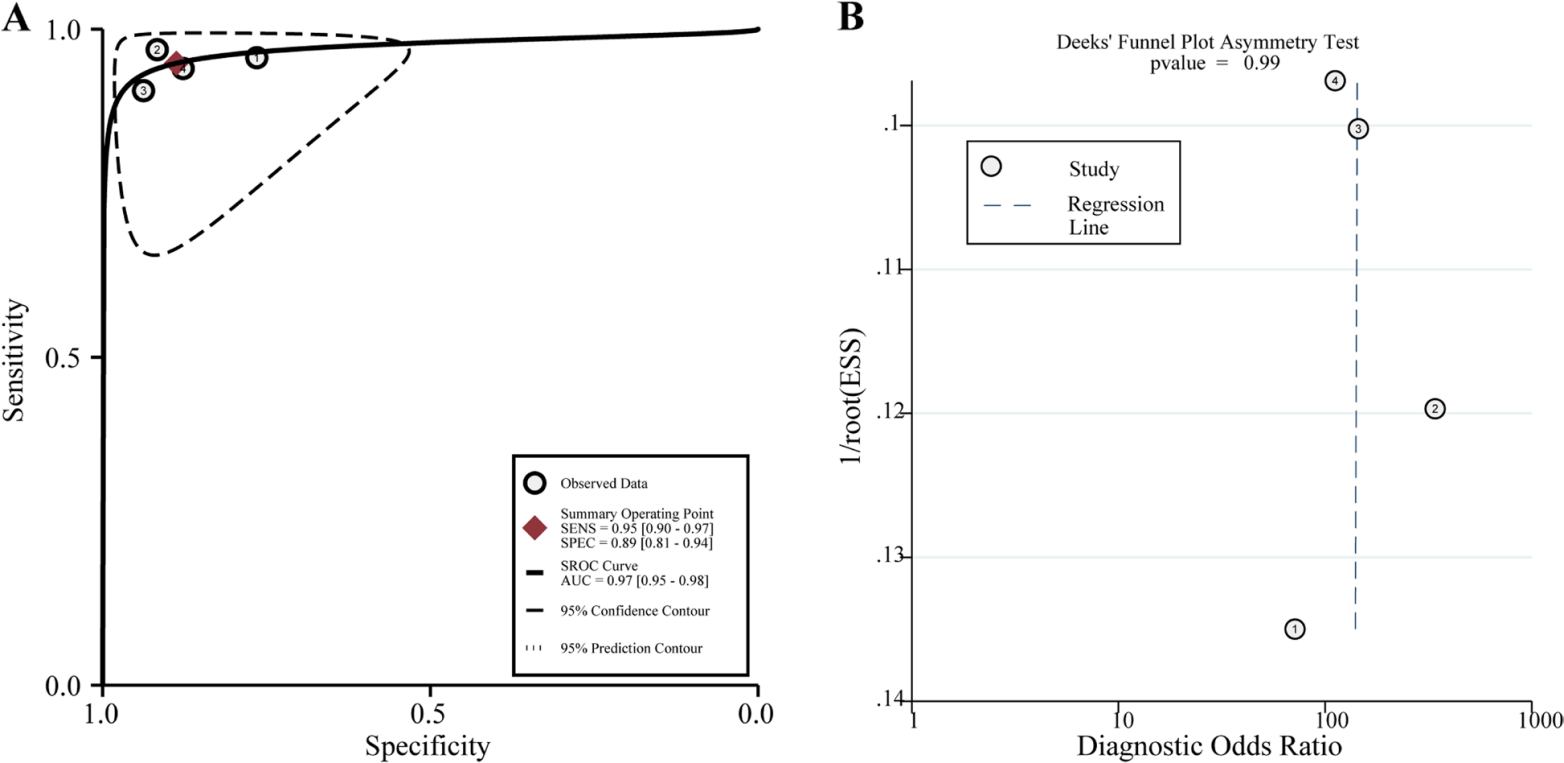
**A** Summary receiver operating characteristic (SROC) curve for diagnosing EEC. **B** Funnel plot for diagnosing EEC

The diagnostic threshold was initially checked, and the Spearman correlation coefficient of ME-NBI combined with EUS for early diagnosis was 0.400 (*P* = 0.60), suggesting no threshold effect. Therefore, the pooled diagnostic values were calculated. Pooled sensitivity, specificity, LR+, LR−, and DOR were 0.947 (95% *CI*, 0.901 to 0.975), 0.894 (95% *CI*, 0.847 to 0.931), 7.989 (95% *CI*, 4.264 to 14.970), 0.066 (95% *CI*, 0.035 to 0.124), and 137.96 (95% *CI*, 60.369 to 315.27), respectively (Figs. 3A and B, 4A and B, and 5). An SROC curve was drawn and showed an AUC value of 0.97 (95% *CI*, 0.95 to 0.98) (Fig. 6A).

The publication bias was assessed, and the Deeks’ funnel plot is shown in Fig. 6B. Statistical tests for small-study effects or publication bias generated a *P*-value of 0.985, indicating the absence of publication bias.

### Meta-analysis of the staging of the invasion depth

**Fig. 7:**
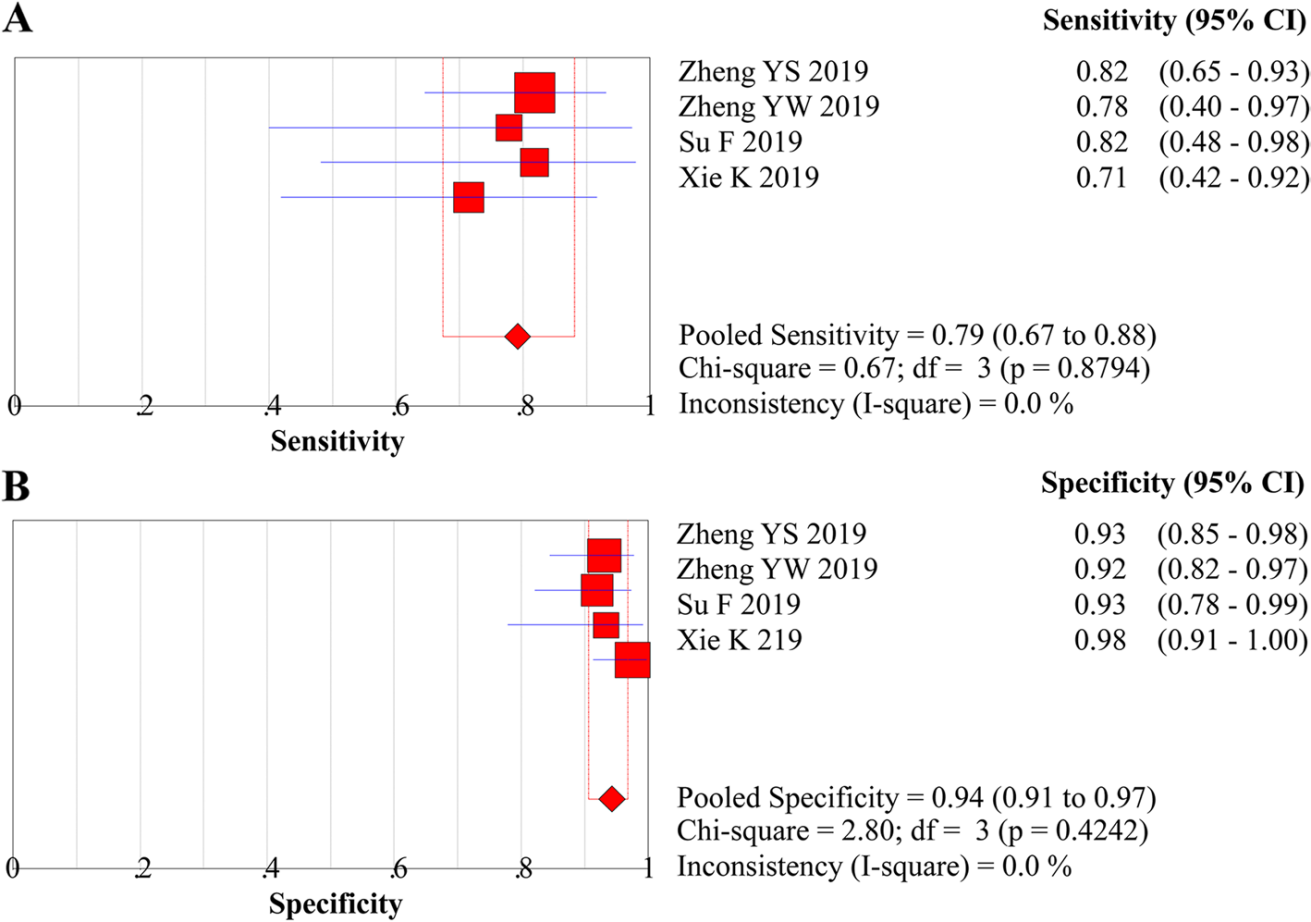
**A** Forest plot of sensitivity for staging of EEC. **B** Forest plot of specificity for staging of EEC

**Fig. 8:**
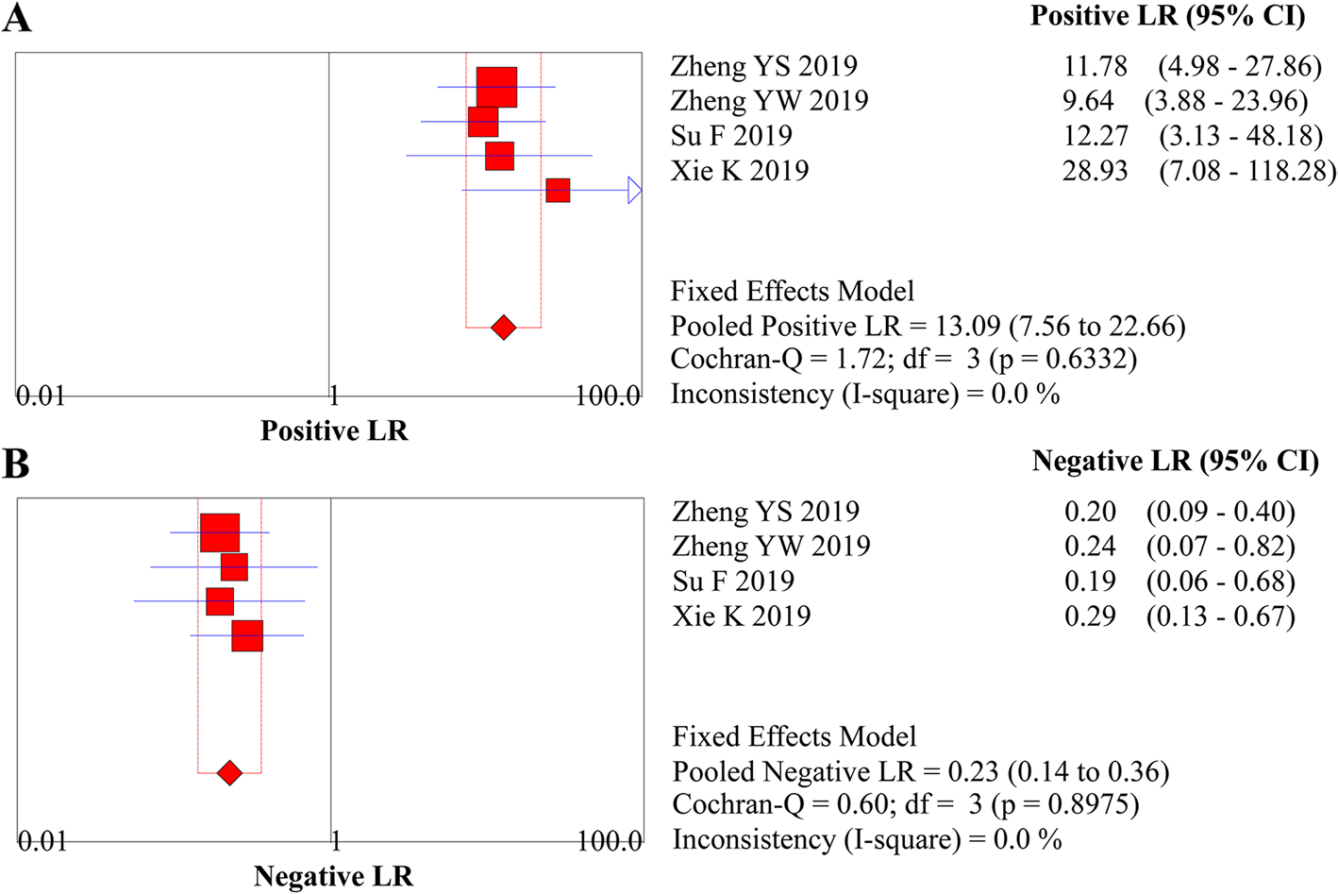
**A** Forest plot of LR+ for staging of EEC. **B** Forest plot of LR− for staging of EEC

**Fig. 9:**
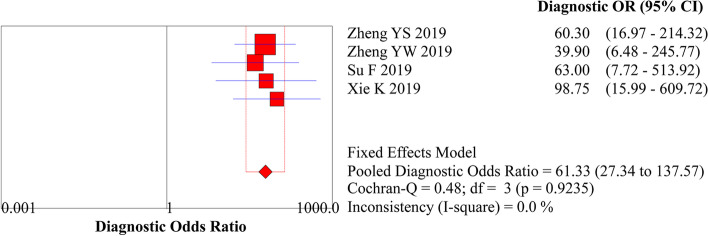
Forest plot of DOR for staging of EEC

**Fig. 10:**
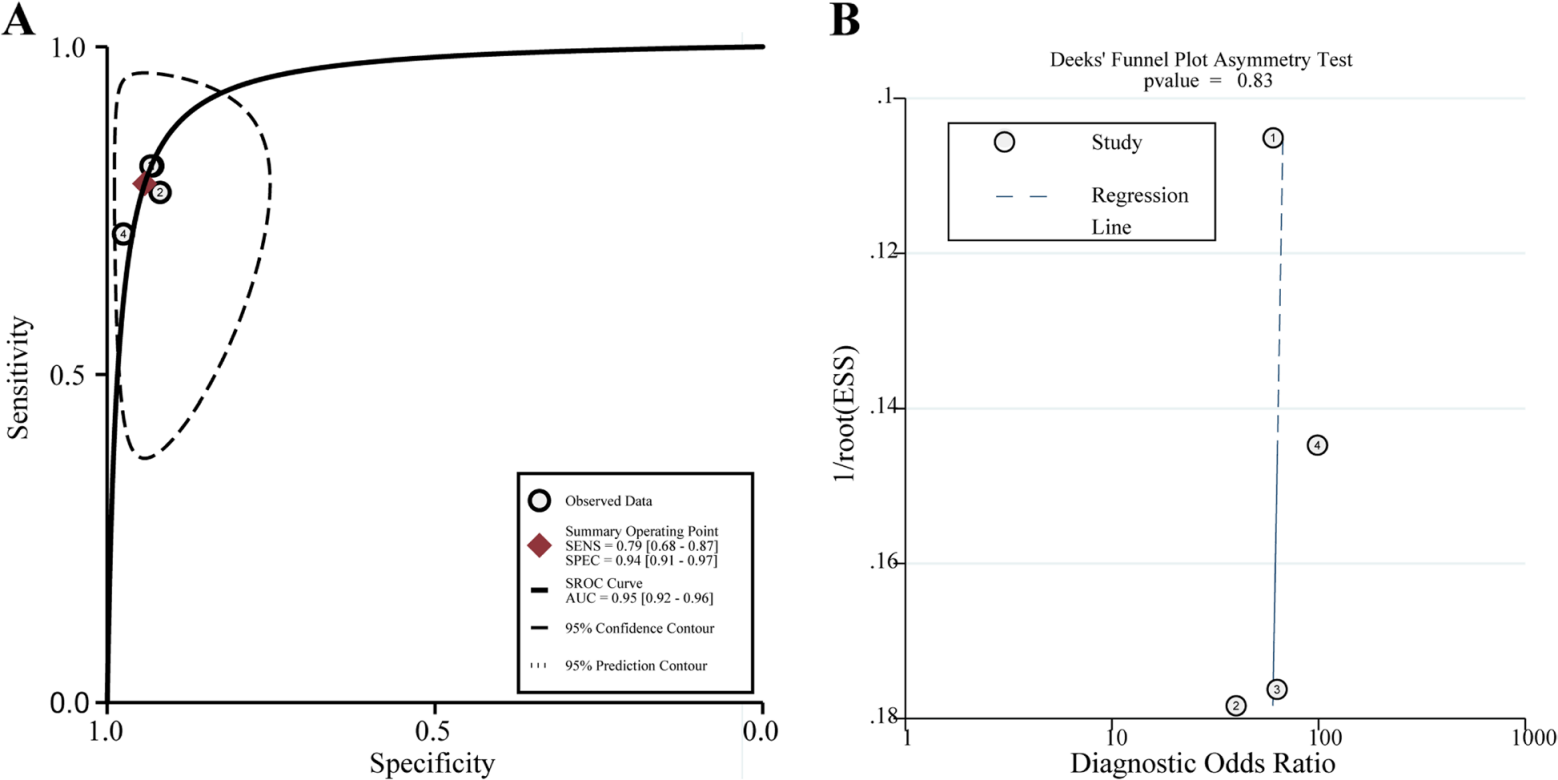
**A** Summary receiver operating characteristic (SROC) curve for staging of EEC. **B** Funnel plot for staging of EEC

The analysis of the diagnostic threshold suggested a Spearman correlation coefficient of 0.316 (*P* = 0.68), indicating the absence of a diagnostic threshold effect when ME-NBI combined with EUS was used to stage the invasion depth. The meta-analysis suggested a pooled sensitivity of 0.791 (95% *CI*, 0.674 to 0.881), specificity of 0.943 (95% *CI*, 0.906 to 0.968), LR+ of 13.087 (95% *CI*, 7.559 to 22.657), LR− of 0.226 (95% *CI*, 0.142 to 0.360), and DOR of 61.332 (95% *CI*, 27.343 to 137.57) (Figs. 7A and B, 8A and B, and 9, respectively). The SROC curve was drawn, and the AUC was 0.95 (95% *CI*, 0.92 to 0.96) (Fig. 10A).

The publication bias was also appraised, and the Deeks’ funnel plot is presented in Fig. 10B. Statistical tests for small-study effects or publication bias generated a *P*-value of 0.832, indicating the absence of publication bias.

### Sensitivity analysis

Significant heterogeneity was detected for pooled specificity (*I*^2^ = 61.0%) and LR+ (*I*^2^ = 61.0%). After the characteristics of four studies were checked, additional sensitivity analyses for the two indices were performed by excluding the study conducted by Wang et al. in 2009 due to insufficient sample size (57 patients). Sensitivity analysis indicated a robust pooled specificity without heterogeneity (0.92; 95% *CI*, 0.87 to 0.95, *I*^2^ = 0.0%). Moreover, the robustness of pooled results in terms of LR+ (pooled estimate, 10.55; 95% *CI*, 6.59 to 16.88; *I*^2^ = 0.0%) after eliminating heterogeneity was also demonstrated in the sensitivity analysis.

## Discussion

### Main findings of the current study

EC is a common malignant tumor with a poor prognosis in the advanced stage [2]. Therefore, it is critically important to detect EC in the early stages and accurately differentiate the invasive depth to provide a reference for the appropriate treatment selection [31]. Several methods based on the endoscopic system have been developed and utilized in clinical practice [10], but no optimal method has been identified. Some studies [12, 13, 27–30] were conducted to investigate the diagnostic accuracy of ME-NBI combined with EUS in the early detection of EEC and differentiation of invasion depth. The present systematic review and meta-analysis were conducted to investigate the pooled estimates, and the results suggested that the combination regimen of ME-NBI plus EUS has achieved high diagnostic accuracy in detecting EEC. Still, the diagnostic value in accurately differentiating the invasion depth requires further investigation.

### Comparison with previous studies

To date, no relevant systematic review and meta-analysis have been published to report the diagnostic accuracy of ME-NBI plus EUS for early diagnosis and accurate differentiation of invasive depth of EC, but several meta-analyses [8, 10, 11, 32, 33] investigated the diagnostic values of ME-NBI or EUS alone for detecting EC, and staging of the invasive depth of EC has been published.

In 2008, Puli et al. investigated the staging accuracy of EUS for EC using meta-analysis [33] and obtained pooled sensitivity of 81.6% (95% *CI*, 77.8 to 84.9) and specificity of 99.4% (95% *CI*, 99.0 to 99.7) for accurately determining the T1 stage. By taking this into account, the authors concluded that EUS had excellent sensitivity and specificity in accurately diagnosing the TN stage of EC.

Thosani et al. performed a meta-analysis to evaluate the diagnostic value of EUS for differentiating the invasiveness of superficial esophageal cancers in 2012 [32]. In this meta-analysis, the pooled sensitivity, specificity, LR+, and LR− for T1a stage were 0.85 (95% *CI*, 0.82 to 0.88), 0.87 (95% *CI*, 0.84 to 0.90), 6.62 (95% *CI*, 3.61 to 12.12), and 0.20 (95% *CI*, 0.14 to 0.30), respectively, and these results for the T1b stage were 0.86 (95% *CI*, 0.82 to 0.89), 0.86 (95% *CI*, 0.83 to 0.89), 5.13 (95% *CI*, 3.36 to 7.82), and 0.17 (95% *CI*, 0.09 to 0.30), respectively. The AUC was more than 0.93 for both mucosal and submucosal lesions. This study concluded that EUS has good diagnostic accuracy for staging superficial esophageal cancers.

In 2017, Morita et al. performed a meta-analysis to determine the diagnostic accuracy of NBI related to Lugol chromoendoscopy for diagnosing esophageal squamous cell carcinoma [8] and found that the pooled sensitivity, specificity, LR+, LR−, and AUC were 94%, 98%, 8.32, 0.16, and 0.956, respectively. Finally, NBI was adequate for establishing high-grade dysplasia and squamous cell carcinoma.

Ishihara et al. also performed a meta-analysis to determine the diagnostic value of various endoscopic imaging modalities for identifying the invasive depth of superficial esophageal squamous cell carcinomas in 2017 [10] and found that ME-NBI had a low LR− [0.08 (95% *CI*, 0.03 to 0.25)] and was regarded as a reliable modality for identifying cancer. EUS had a high LR+ [17.6 (95% *CI*, 6.7 to 46.3)] and was considered a reliable method for determining the invasive depth of cancer. Therefore, the effectiveness of ME-NBI and EUS should be considered in patients with esophageal squamous cell carcinoma.

In 2018, Yu et al. investigated the diagnostic accuracy of ME-NBI for esophageal squamous cell carcinoma [11] and found that the pooled sensitivity, specificity, LR+, LR−, and AUC of ME-NBI for diagnosing esophageal squamous cell carcinoma were 0.90 (95% *CI*, 0.71 to 0.97), 0.90 (95% *CI*, 0.80 to 0.95), 6.74 (95% *CI*, 3.52 to 712.89), 0.20 (95% *CI*, 0.10 to 0.42), and 0.95, respectively. However, the pooled sensitivity, specificity, LR+, LR−, and DOR of EUS for differentiating the invasion depth were 0.83 (95% *CI*, 0.71 to 0.94), 0.88 (95% *CI*, 0.76 to 0.99), 7.30 (95% *CI*, 4.18 to 12.72), 0.19 (95% *CI*, 0.10 to 0.38), and 36.71 (95% *CI*, 12.61 to 106.89), respectively. So, ME-NBI and EUS were considered reliable methods for detecting esophageal squamous cell carcinoma and confirming the invasion depth of cancer.

Compared with these previous meta-analyses with various diagnostic accuracies of ME-NBI or EUS alone in diagnosing and differentiating the invasive depth of EC, the present study firstly investigated the diagnostic accuracy of ME-NBI plus EUS for diagnosing EEC and staging the invasive depth of EC. Our results further supported the potential conclusion proposed by Ishihara et al. and suggested that the combination of ME-NBI plus EUS was preferably used for the early detection of EC. However, the value for accurate differentiation of invasive depth of EC should be further investigated as only acceptable diagnostic yields were obtained.

### Limitations of the current study

The estimated pooled diagnostic accuracy of ME-NBI combined with EUS was done by pooling seven eligible studies, showing that ME-NBI plus EUS achieved higher diagnostic accuracy. However, some limitations of the present study should be considered. First, all eligible studies were carried out by Chinese authors, and thus, the results should be cautiously interpreted in the clinical scenario of western countries. Second, some factors (such as equipment and experience of endoscopists) have been confirmed to negatively affect the pooled results, but subgroup analysis or sensitivity analysis was not performed to test the robustness of pooled diagnostic accuracy due to the limited number of eligible studies for individual outcomes. Therefore, further studies are warranted to establish the value of ME-NBI plus EUS for EEC. Third, although seven eligible studies were included in the final analysis, only four with a small sample size were incorporated in the pooled analysis for individual outcomes. Thus, pooled diagnostic accuracy should be required for cautious interpretation. Fourth, the overall quality of all eligible studies was low to moderate, impairing the reliability of the pooled results. Therefore, carrying out high quality is suggested to clarify the diagnostic accuracy of ME-NBI plus EUS for the early diagnosis and accurate staging of EEC. Fifth, significant heterogeneity was detected for specificity and pooled LR+. Therefore, a study without a sufficient sample size was excluded from performing sensitivity analyses and demonstrated the robustness of all pooled results of the two indices. Nevertheless, more studies with adequate sample sizes are warranted to determine further the diagnostic yield of ME-NBI plus EUS for diagnosis and differentiation of EC.

ME-NBI combined with EUS provides a high diagnostic rate for the early identification of EC, but its value for the accurate identification of the EC invasion depth is still conflicting. More studies with large samples and high quality should be performed to confirm the diagnostic accuracy of ME-NBI combined with EUS, as the insufficient number of eligible studies, accumulated sample size, and inadequate quality of the included studies might impair the reliability and robustness of the pooled results. Moreover, the confounding factors should be investigated to determine the pure diagnostic value of ME-NBI plus EUS for early diagnosis of EC and accurate differentiation of the invasive depth of EC to provide a reference for selecting an appropriate treatment regimen.

## Supplementary Information

**Additional file 1: Table S1.** PubMed search strategy. **Table S2.** Embase search strategy. **Table S3.** Cochrane library search strategy. **Table S4.** CNKI search strategy (in Chinese).

## Data Availability

All data generated or analyzed during this study are included in this published article.
