# Supplementary material for: ME-NBI combined with endoscopic ultrasonography for diagnosing and staging the invasion depth of early esophageal cancer: a diagnostic meta-analysis
Source: World J Surg Oncol. 2022 Oct 17;20:343. doi: 10.1186/s12957-022-02809-6 (PMC9575268; doi:10.1186/s12957-022-02809-6)
Supplement: Supplementary file 1 — Additional file 1: Table S1. PubMed search strategy. Table S2. Embase search strategy. Table S3. Cochrane library search strategy. Table S4. CNKI search strategy (in Chinese). [file 12957_2022_2809_MOESM1_ESM.doc]

Table S1. PubMed search strategy

| Search number | Query |
| --- | --- |
| 11 | ((((((("Endoscopy, Digestive System"[Mesh]) OR "Endoscopy, Gastrointestinal"[Mesh]) OR "Endoscopy"[Mesh]) OR "Gastroscopy"[Mesh]) OR ((((((((((Digestive System Endoscop*[Title/Abstract]) OR (Digestive System Endoscopic Surgical Procedur*[Title/Abstract])) OR (Digestive System Endoscopic Surge*[Title/Abstract])) OR (Esophagogastroduodenoscop*[Title/Abstract])) OR (Surgical Endoscop*[Title/Abstract])) OR (endoscop*[Title/Abstract])) OR (Endoscopic Surgical Procedur*[Title/Abstract])) OR (Gastroscop*[Title/Abstract])) OR (Gastroscopic Surgical Procedur*[Title/Abstract])) OR (Gastroscopic Surger*[Title/Abstract]))) AND (("Endosonography"[Mesh]) OR ((((((((Endosonograph*[Title/Abstract]) OR (Echo Endoscopi*[Title/Abstract])) OR (Echo-Endoscopy[Title/Abstract])) OR (Echo Endoscopy[Title/Abstract])) OR (Echo-Endoscopies[Title/Abstract])) OR (Ultrasonic Endoscopi*[Title/Abstract])) OR (Endoscopic Ultrasonography[Title/Abstract])) OR (Endoscopic Ultrasonographies[Title/Abstract])))) AND ((narrow banding imaging[Title/Abstract]) OR (NBI[Title/Abstract]))) AND (("Esophageal Neoplasms"[Mesh]) OR ((((Esophageal Neoplasm*[Title/Abstract]) OR (Esophagus Neoplasm*[Title/Abstract])) OR (Esophagus Cancer*[Title/Abstract])) OR (Esophageal Cancer*[Title/Abstract]))) |
| 10 | ("Esophageal Neoplasms"[Mesh]) OR ((((Esophageal Neoplasm*[Title/Abstract]) OR (Esophagus Neoplasm*[Title/Abstract])) OR (Esophagus Cancer*[Title/Abstract])) OR (Esophageal Cancer*[Title/Abstract])) |
| 9 | (((Esophageal Neoplasm*[Title/Abstract]) OR (Esophagus Neoplasm*[Title/Abstract])) OR (Esophagus Cancer*[Title/Abstract])) OR (Esophageal Cancer*[Title/Abstract]) |
| 8 | "Esophageal Neoplasms"[Mesh] |
| 7 | (narrow banding imaging[Title/Abstract]) OR (NBI[Title/Abstract]) |
| 6 | ("Endosonography"[Mesh]) OR ((((((((Endosonograph*[Title/Abstract]) OR (Echo Endoscopi*[Title/Abstract])) OR (Echo-Endoscopy[Title/Abstract])) OR (Echo Endoscopy[Title/Abstract])) OR (Echo-Endoscopies[Title/Abstract])) OR (Ultrasonic Endoscopi*[Title/Abstract])) OR (Endoscopic Ultrasonography[Title/Abstract])) OR (Endoscopic Ultrasonographies[Title/Abstract])) |
| 5 | (((((((Endosonograph*[Title/Abstract]) OR (Echo Endoscopi*[Title/Abstract])) OR (Echo-Endoscopy[Title/Abstract])) OR (Echo Endoscopy[Title/Abstract])) OR (Echo-Endoscopies[Title/Abstract])) OR (Ultrasonic Endoscopi*[Title/Abstract])) OR (Endoscopic Ultrasonography[Title/Abstract])) OR (Endoscopic Ultrasonographies[Title/Abstract]) |
| 4 | "Endosonography"[Mesh] |
| 3 | (((("Endoscopy, Digestive System"[Mesh]) OR "Endoscopy, Gastrointestinal"[Mesh]) OR "Endoscopy"[Mesh]) OR "Gastroscopy"[Mesh]) OR ((((((((((Digestive System Endoscop*[Title/Abstract]) OR (Digestive System Endoscopic Surgical Procedur*[Title/Abstract])) OR (Digestive System Endoscopic Surge*[Title/Abstract])) OR (Esophagogastroduodenoscop*[Title/Abstract])) OR (Surgical Endoscop*[Title/Abstract])) OR (endoscop*[Title/Abstract])) OR (Endoscopic Surgical Procedur*[Title/Abstract])) OR (Gastroscop*[Title/Abstract])) OR (Gastroscopic Surgical Procedur*[Title/Abstract])) OR (Gastroscopic Surger*[Title/Abstract])) |
| 2 | (((((((((Digestive System Endoscop*[Title/Abstract]) OR (Digestive System Endoscopic Surgical Procedur*[Title/Abstract])) OR (Digestive System Endoscopic Surge*[Title/Abstract])) OR (Esophagogastroduodenoscop*[Title/Abstract])) OR (Surgical Endoscop*[Title/Abstract])) OR (endoscop*[Title/Abstract])) OR (Endoscopic Surgical Procedur*[Title/Abstract])) OR (Gastroscop*[Title/Abstract])) OR (Gastroscopic Surgical Procedur*[Title/Abstract])) OR (Gastroscopic Surger*[Title/Abstract]) |
| 1 | ((("Endoscopy, Digestive System"[Mesh]) OR "Endoscopy, Gastrointestinal"[Mesh]) OR "Endoscopy"[Mesh]) OR "Gastroscopy"[Mesh] |

Table S2. Embase search strategy

| No. | Query |
| --- | --- |
| #11 | #3 AND #6 AND #7 AND #10 |
| #10 | #8 OR #9 |
| #9 | 'esophagus tumor'/exp |
| #8 | 'esophageal neoplasm':ti,ab,kw OR 'esophageal neoplasms':ti,ab,kw OR 'esophagus neoplasm':ti,ab,kw OR 'esophagus neoplasms':ti,ab,kw OR 'esophagus cancer':ti,ab,kw OR 'esophagus cancers':ti,ab,kw OR 'esophageal cancer':ti,ab,kw OR 'esophageal cancers':ti,ab,kw OR 'esophagus tumor':ti,ab,kw OR 'esophagus tumors':ti,ab,kw OR 'esophageal tumor':ti,ab,kw OR 'esophageal tumors':ti,ab,kw |
| #7 | 'narrow banding imaging':ti,ab,kw OR nbi:ti,ab,kw |
| #6 | #4 OR #5 |
| #5 | 'endoscopic ultrasonography'/exp |
| #4 | endosonography:ti,ab,kw OR endosonographies:ti,ab,kw OR 'echo endoscopy':ti,ab,kw OR 'echo endoscopies':ti,ab,kw OR 'ultrasonic endoscopies':ti,ab,kw OR 'ultrasonic endoscopy':ti,ab,kw OR 'endoscopic ultrasonography':ti,ab,kw OR 'endoscopic ultrasonographies':ti,ab,kw |
| #3 | #1 OR #2 |
| #2 | 'digestive tract endoscopy'/exp OR 'gastrointestinal endoscopy'/exp OR 'endoscopy'/exp OR 'gastroscopy'/exp |
| #1 | 'digestive system endoscopy':ti,ab,kw OR 'digestive system endoscopies':ti,ab,kw OR 'digestive system endoscopic surgical procedure':ti,ab,kw OR 'digestive system endoscopic surgical procedures':ti,ab,kw OR 'digestive system endoscopic surgery':ti,ab,kw OR 'digestive system endoscopic surgeries':ti,ab,kw OR esophagogastroduodenoscopy:ti,ab,kw OR esophagogastroduodenoscopies:ti,ab,kw OR 'surgical endoscopy':ti,ab,kw OR 'surgical endoscopies':ti,ab,kw OR endoscopy:ti,ab,kw OR endoscopies:ti,ab,kw OR 'endoscopic surgical procedure':ti,ab,kw OR 'endoscopic surgical procedures':ti,ab,kw OR gastroscopy:ti,ab,kw OR gastroscopies:ti,ab,kw OR 'gastroscopic surgery':ti,ab,kw OR 'gastroscopic surgeries':ti,ab,kw |

Table S3. Cochrane library search strategy

| No. | Query |
| --- | --- |
| #1 | (digestive system endoscop*):ti,ab,kw OR (Digestive System Endoscopic Surgical Procedur*):ti,ab,kw OR (Digestive System Endoscopic Surge*):ti,ab,kw OR (Esophagogastroduodenoscop*):ti,ab,kw OR (Surgical Endoscop*):ti,ab,kw (Word variations have been searched) |
| #2 | (digestive system endoscop*):ti,ab,kw OR (Endoscopic Surgical Procedur*):ti,ab,kw OR (Gastroscop*):ti,ab,kw OR (Gastroscopic Surgical Procedur*):ti,ab,kw OR (Gastroscopic Surger*):ti,ab,kw (Word variations have been searched) |
| #3 | #1 OR #2 |
| #4 | MeSH descriptor: [Endoscopy, Gastrointestinal] explode all trees |
| #5 | MeSH descriptor: [Endoscopy] explode all trees |
| #6 | MeSH descriptor: [Gastroscopy] explode all trees |
| #7 | MeSH descriptor: [Endoscopy, Digestive System] explode all trees |
| #8 | #3 OR #4 OR #5 OR #6 OR #7 |
| #9 | (Endosonograph*):ti,ab,kw OR (Echo Endoscopi*):ti,ab,kw OR (Echo-Endoscop*):ti,ab,kw OR (Echo-Endoscopi*):ti,ab,kw OR (Ultrasonic Endoscopi*):ti,ab,kw (Word variations have been searched) |
| #10 | (Endoscopic Ultrasonography):ti,ab,kw OR (Endoscopic Ultrasonographies):ti,ab,kw (Word variations have been searched) |
| #11 | #9 OR #10 |
| #12 | MeSH descriptor: [Endosonography] explode all trees |
| #13 | #11 OR #12 |
| #14 | (narrow banding imaging):ti,ab,kw OR (NBI):ti,ab,kw (Word variations have been searched) |
| #15 | (Esophageal Neoplasm*):ti,ab,kw OR (Esophagus Neoplasm*):ti,ab,kw OR (Esophagus Cancer*):ti,ab,kw OR (Esophageal Cancer*):ti,ab,kw OR (Esophageal tumor*):ti,ab,kw (Word variations have been searched) |
| #16 | MeSH descriptor: [Esophageal Neoplasms] explode all trees |
| #17 | #15 OR #16 |
| #18 | #8 AND #13 AND #14 AND #17 |

**Table S4. CNKI sea**rch strategy (in Chinese)

| 检索主题：ME-NBI-EUS |
| --- |
| 检索范围：中国学术期刊网络出版总库,中国博士学位论文全文数据库,中国优秀硕士学位论文全文数据库,中国重要会议论文全文数据库,国际会议论文全文数据库,中国重要报纸全文数据库,中国学术辑刊全文数据库,外文期刊,国际会议 |
| 检索年限：不限 |
| 检索式A:( (主题=胃镜 或者 题名=胃镜 或者 v_subject=中英文扩展(胃镜) 或者 title=中英文扩展(胃镜)) 或者 (主题=内镜 或者 题名=内镜 或者 v_subject=中英文扩展(内镜) 或者 title=中英文扩展(内镜)) ) 并且 (主题=放大 或者 题名=放大 或者 v_subject=中英文扩展(放大) 或者 title=中英文扩展(放大)) 并且 ( (主题=中英文扩展(NBI) 或者 题名=中英文扩展(NBI) 或者 v_subject=NBI 或者 title=NBI) 或者 (主题=窄带成像 或者 题名=窄带成像 或者 v_subject=中英文扩展(窄带成像) 或者 title=中英文扩展(窄带成像)) ) 并且 (主题=超声 或者 题名=超声 或者 v_subject=中英文扩展(超声) 或者 title=中英文扩展(超声)) 并且 ( (主题=食管 或者 题名=食管 或者 v_subject=中英文扩展(食管) 或者 title=中英文扩展(食管)) 或者 (主题=食道 或者 题名=食道 或者 v_subject=中英文扩展(食道) 或者 title=中英文扩展(食道)) ) 并且 ( (主题=癌 或者 题名=癌 或者 v_subject=中英文扩展(癌) 或者 title=中英文扩展(癌)) 或者 (主题=肿瘤 或者 题名=肿瘤 或者 v_subject=中英文扩展(肿瘤) 或者 title=中英文扩展(肿瘤)) ) (模糊匹配) |
